# Laryngeal oncocytic cystadenoma and sudden death

**DOI:** 10.1007/s12024-022-00530-0

**Published:** 2022-09-22

**Authors:** John D. Gilbert, Roger W. Byard

**Affiliations:** 1 Pathology, Forensic Science SA, SA Adelaide, Australia; 2 School of Biomedicine, The University of Adelaide, Frome Road, Adelaide, South Australia 5005 Australia

**Keywords:** Oncocytic cyst, Oncocytic cystadenoma, Larynx, Stridor, Airway obstruction; Sudden death

## Abstract

An 86-year-old woman with Alzheimer disease collapsed in her nursing home and was not able to be resuscitated. At autopsy, the major findings were in the larynx where a pedunculated oncocytic cystadenoma had occluded the glottis. Oncocytic cysts or cystadenomas of the larynx are rare histologically benign lesions that account for only 0.1–1% of laryngeal lesions. While the usual presentation is of a sensation of a mass in the throat, hoarseness, or stridor, very occasionally, there may be acute airway compromise and sudden death. Oncocytic cystadenoma should, therefore, be included in the differential diagnosis of potentially lethal obstructive laryngeal lesions.

## Case report

An 86-year-old woman with Alzheimer disease was resident in a nursing home. She had a past history of Takatsubo cardiomyopathy, uterine prolapse, depression, and a right frontal lobe meningioma with epilepsy. She died after being found collapsed on a corridor floor.

**Fig. 1:**
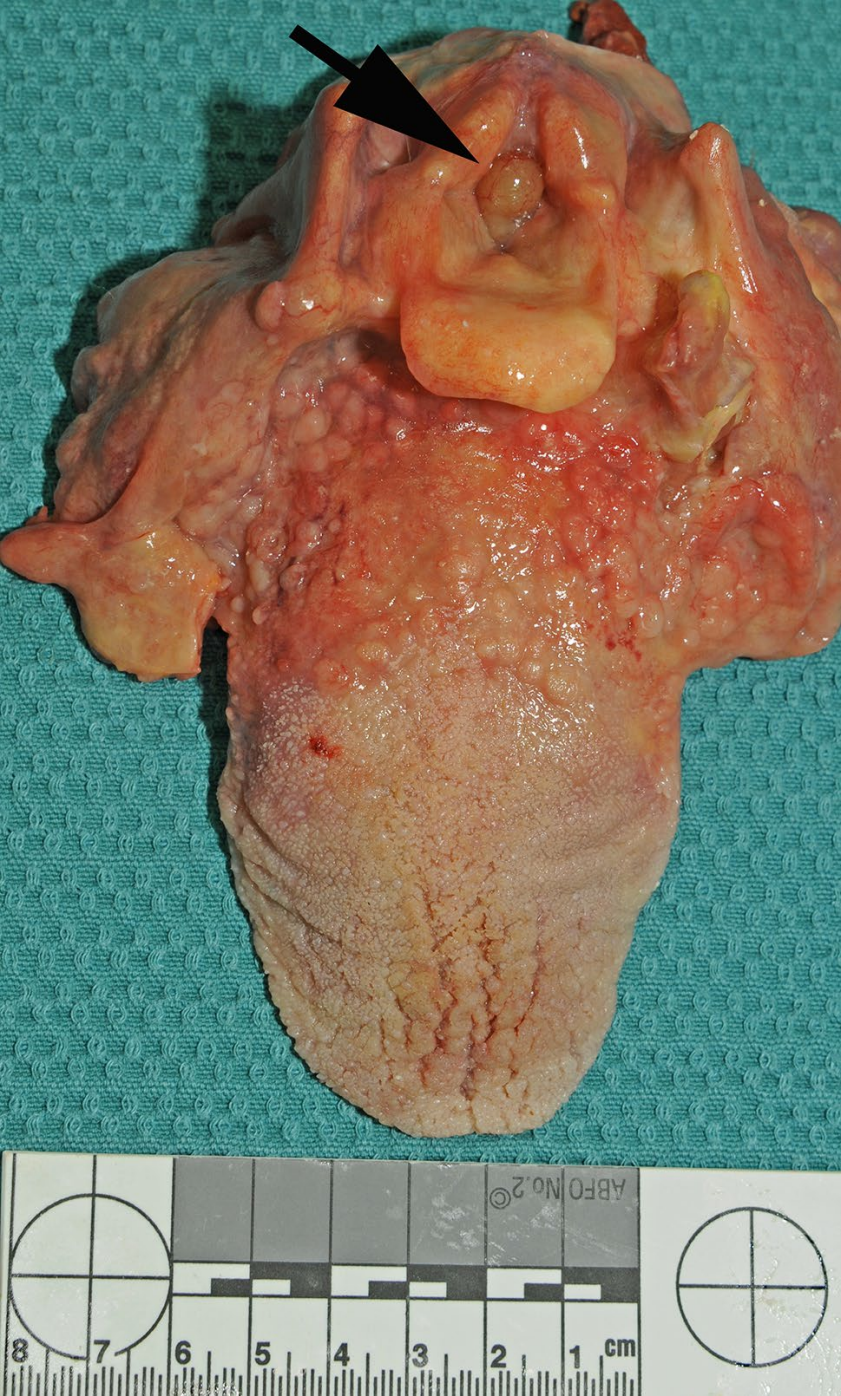
A view of the epiglottis and laryngeal inlet from above revealing an obstructive lesion at the glottis (arrow)

**Fig. 2:**
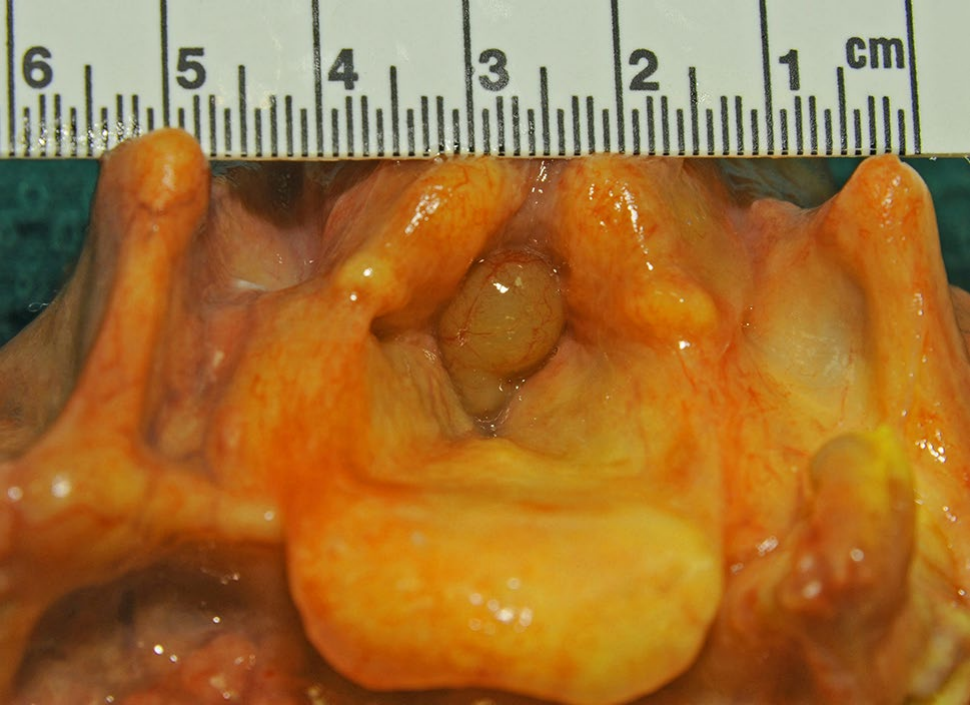
A closer view of the polypoidal mass occluding the upper airway

**Fig. 3:**
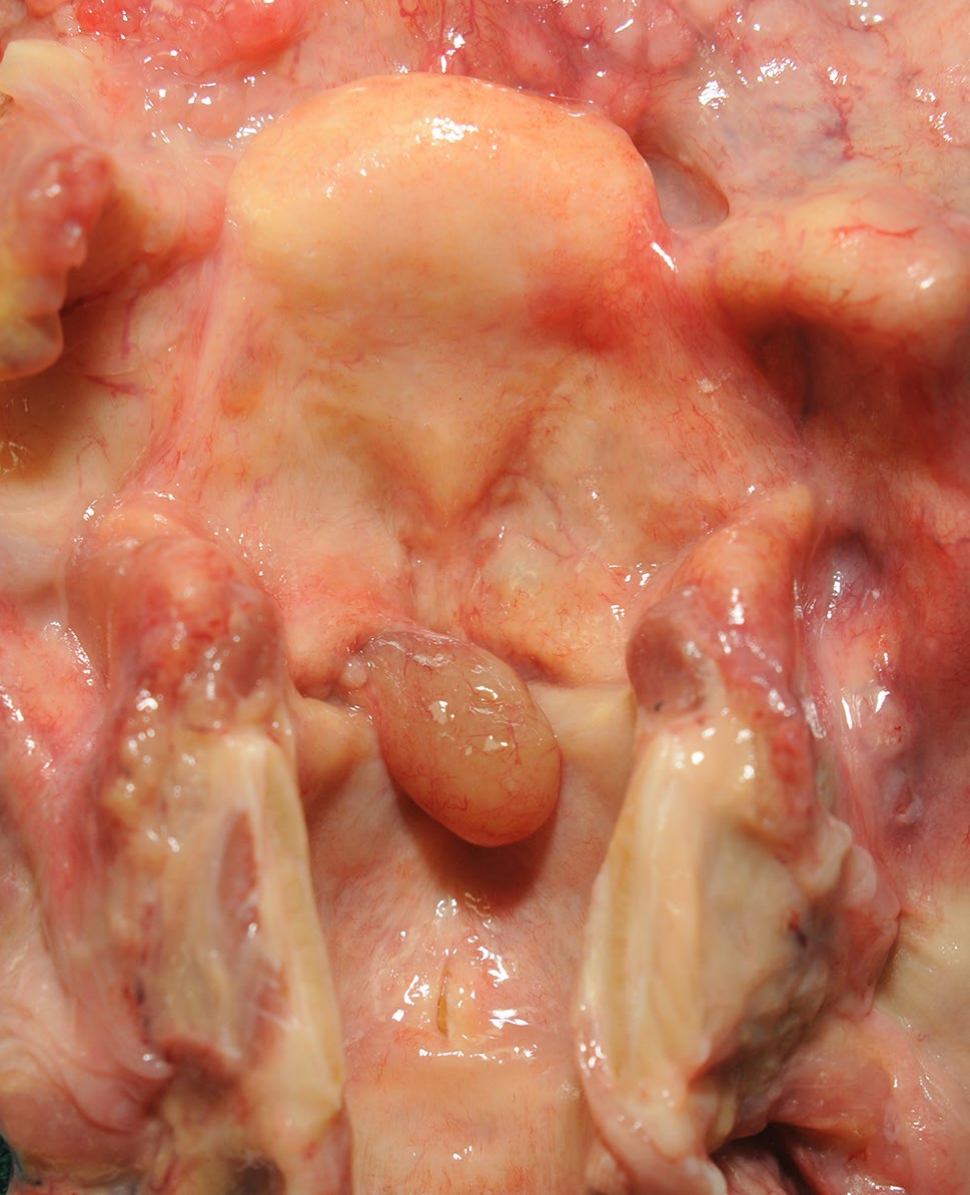
Opening of the posterior wall of the larynx to more clearly show the polyp

**Fig. 4:**
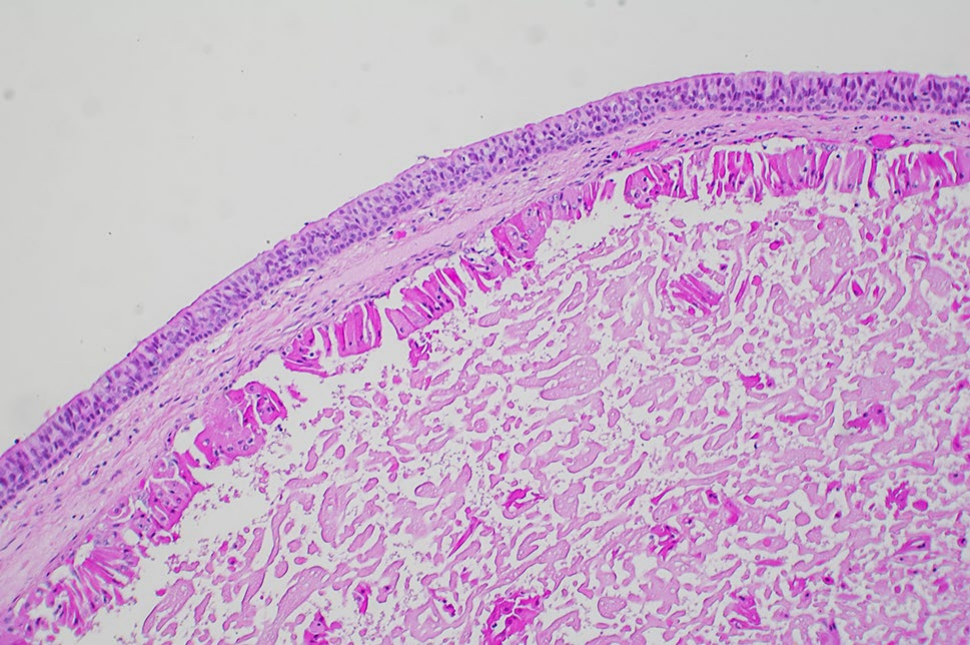
Histological sections of the polyp revealed an external covering of orderly pseudostratified columnar epithelium overlying a thin fibrous wall lined by large and tall columnar cells with granular eosinophilic cytoplasm typical of an oncocytic cystadenoma (hematoxylin & eosin × 150)

At autopsy, the major findings were in the larynx where a pedunculated 15 × 8 × 7-mm pale brown translucent cyst arose from the mid-portion of the left laryngeal ventricle (between the left false cord and left vocal cord) and projected upwards over the base of the epiglottis (Figs. 1, 2, and 3). The remainder of the larynx appeared normal. Histologically the cyst was composed of a thin fibrous wall lined internally by large and tall columnar cells with granular eosinophilic cytoplasm, with relatively small nuclei located towards the apex of the cells. There were occasional fibrovascular papillary infoldings lined by similar cells. The cyst contained amorphous mildly eosinophilic secretions. An outer layer of orderly pseudostratified columnar epithelium was present. No malignancy was identified and the appearances were those of a laryngeal oncocytic cystadenoma (Fig. 4).

Other findings included mild pulmonary emphysema and mild left ventricular hypertrophy and dilatation of the heart with no significant coronary atherosclerosis. Neuropathological examination of the brain identified a right frontal meningioma (WHO Grade I, meningothelial meningioma) measuring 46 × 28 mm which had compressed but not invaded the right frontal lobe of the brain. Only minor cortical reactive changes were seen in the brain adjacent to the tumor. Elsewhere, the brain showed changes in keeping with Alzheimer disease. There were no features of raised intracranial pressure or of tentorial or cerebellar tonsillar herniation. There were no other organic diseases present which could have caused or contributed to death and there was no trauma.

Toxicological evaluation of blood revealed no alcohol, with a greater than therapeutic level of carbamazepine and a therapeutic level of paliperidone, a metabolite of risperidone. No other common drugs were detected.

Death was attributed to upper airway obstruction by a laryngeal oncocytic cystadenoma against a background of Alzheimer disease and epilepsy.

## Discussion

A wide variety of lesions and conditions may result in acute upper airway obstruction in children and adults [1]. These may be extrinsic to the airway causing critical narrowing of the air passages from external pressure as in cases of benign goiter, malignancy, or hemorrhage into soft tissues [1, 2]. Alternatively, blockage may be intrinsic from a medical condition which could be congenital, as in lingual thyroglossal duct cysts, or acquired such as laryngeal carcinoma or infections such as Ludwig angina or diphtheria [3–6].

Critical swelling may also be caused by anaphylaxis or may be due to edema from burns [7]. One of the more common scenarios in medicolegal settings is the so-called café coronary syndrome when the upper airway is blocked by poorly-masticated food, often in demented or intoxicated individuals [8]. Inhalation of foreign material into the upper airway may also occur in industrial accidents where burial in soft material has occurred [9].

Benign tumors may also cause acute obstruction sometimes resulting in sudden death, as in a case of a polypoidal hemangioma in a previously-well 42-year-old man [1]. Polypoidal lesions such as inflammatory polyps, papillomas, polypoidal basosquamous carcinomas of the epiglottis, pedunculated schwannomas of the aryepiglottic fold, and pedunculated cavernous hemangiomas may be a particular problem in adults as their mobility and shape may predispose to sudden airway occlusion [1, 10, 11]. In the current case, it is likely that a laryngeal oncocytic cystadenoma sitting at the base of the epiglottis caused critical airway obstruction. It is possible that underlying dementia had prevented the decedent from reporting symptoms prior to death.

Oncocytic cysts or cystadenomas of the larynx are rare histologically benign lesions that account for only 0.1–1% of laryngeal lesions [12]. They were first described independently by both Koschier and Schaffer in 1897, and up to 2007, there were only 150 published cases in the literature [13–15]. The lining oncocytes represent hypertrophic epithelial cells that contain numerous mitochondria resulting in the characteristic abundant eosinophilic cytoplasm [16] as can be seen in Fig. 4. The lesions usually arise in the supraglottic larynx, the ventricular folds, the laryngeal ventricles, or the aryepiglottic folds. Very rarely they may be subglottic and multiple [17, 18]. Most often, there is a history of smoking with the lesions being diagnosed in women over the age of 60 years [13]. Treatment is by local excision with follow-up for recurrences [12].

The usual presentation is of a sensation of a mass in the throat, hoarseness, or stridor. Very occasionally, there may, however, be acute obstructive symptoms and as the current case shows death [19]. This case has, therefore, demonstrated a rare tumor of the larynx with an even less common presentation, that of sudden death. Oncocytic cystadenoma should be included in the differential diagnosis of potentially lethal obstructive laryngeal lesions.
